# A New Edition of the Reference Hand-book of the Medical Sciences

**Published:** 1900-08

**Authors:** 


### Books and Magazines.

### A New Edition of the Reference Hand-book of the Medical Sciences.

NEW YORK, June 25, 1900.

*Editor Texas Medical Journal.*

DEAR SIR: Twenty year ago, in conjunction with Dr. Albert H. Buck, of this city, plans were laid which resulted in the production during the ensuing five years of what has been unquestionably the most popular medical work ever printed in the English language. At that time the association of numbers of writers in the making of any medical work was entirely new. The result of the combination of so large a number of prominent authorities as Dr. Buck succeeded in interesting in the Reference Hand-book of the

Medical Sciences, was such an unqualified success, as to have inaugurated an entirely new system in the production of encyclopædic medical works. While, however, such an organization was absolutely essential in a work covering a field so wide, and demanding such a great variety of medical erudition, the mistake has been made by other publishers emulous of similar success, of flooding the country with numerous other publications, not justified by the demands of the times or the needs of the profession.

It might almost be truthfully asserted that a physician who possessed a set of the Reference Hand-book was well equipped for the practice of his profession, so far as books were concerned, with the purchase only of occasional monographs upon such specialities as he desired to give extended study to. Never in all the score of years past, have we heard from any one who possessed a copy of this work anything but almost extravagant commendation. Of all the books in their library, this has been the one to which constant daily reference is made. It is made to cover the entire field of medicine, surgery, and their allied sciences, and we believe it has satisfied every reasonable expectation.

The time has come, however, when it has seemed necesary that the work should be thoroughly brought up to the times. After a very thorough and careful investigation of the work, Dr. Buck came to the conclusion that it would be necessary entirely to rewrite it from beginning to end, and although involving an almost incomprehensible amount of detail and labor, it would also be necessary, to satisfy the just requirements of the profession at this day, to reorganize a new staff of writers, and begin the work anew, almost as if it had never before been undertaken.

If you will kindly look over the prospectus of the new Reference Hand-book which accompanies this letter, we think you will see at once that no other medical book ever published can approach this in its magnificent organization and complete corps of writers.

Kindly note the list of department advisers, and then the long list of between three and four hundred writers. When complete this list will include in the neighborhood of 500 contributors working under their direction and supervision. The size of the page of the new Reference Hand-book is practically the same as that of the original edition, experience having shown that this could not be materially improved upon.

In the matter of illustrations, however, we should be pleased to have you compare those we are now making with similar ones of the original edition, showing the great advance made in the past twenty years in the fineness and elegance of mechanical execution, based in almost all cases upon similar advance in photographic technique. The paper used in the new Reference Hand-book, in order to bring out the beauty of the illustrations, will necessarily be of much higher finish than before.

A greatly increased number of exquisite chromo-lithographic plates are being prepared. The first volume contains no less than fourteen of these.

In thus calling your attention to this new work, we feel a pride, wholly pardonable we think, in referring to the past history of our house, established nearly one hundred years ago, in that our imprint upon the title page of any work has never failed to stamp the publication bearing it as worthy of the most implicit confidence of the medical profession. It has always been our ambition to give in all our publications more than we promise, and it is to the recognition of this fact that is due, in no small degree, the confidence of the medical profession in the value of all of our publications, and the unparalleled patronage which has been extended to them.

In issuing then this new and standard work, we bespeak your most efficient co-operation in making of it, as before, such an unqualified success as will justify the expenditure of this nearly two hundred thousand dollars, which it will cost to place it upon the market.

We propose to send the volumes of the new edition of the Reference Hand-book to you for review as they are issued, and shall be very pleased if you feel disposed at this time not to print this communication, as it stands, but possibly using it as a suggestive foundation to make an early announcement of our intentions. The first volume will be issued about the middle of August next.

With kind regards, we are,

Very sincerely your friends,

William Wood & Co.

---

The Irrigation Treatment of Gonorrhoea—Its Local Treatment and Sequelae.—By Ferd. C. Valentine, M. D., Professor of Genito-Urinary Diseases, New York School of Clinical Medicine; Genito-Urinary Surgeon, West Side German Dispensary; Genito-Urinary Consultant to the United Hebrew Charities in the Metropolitan Hospital and Dispensary, etc., etc. One volume, profusely illustrated. Muslin, $2.00, net. Illustrated by fifty-seven engravings. William Wood & Company, New York.

We are told, "The poor are always with us." So it is with gonorrhœa; hence the importance and necessity for the physician to thoroughly understand this most important subject; not merely for the good of the patient himself, but also for the benefit of the women and children who are oftentimes innocent victims.

The irrigation treatment of gonorrhœa is unquestionably the most rational treatment, and the one which offers the least percentage of complications and sequelæ; hence the very great value of this work.

The author understood the necessity of a work upon this subject for the general practitioners all over the country, who must by necessity become his own specialist upon venereal diseases, and this valuable work is of inestimable value for the busy physician. It is divested of all mooted questions and speculative theories, and deals with the common sense, practical side of the question, ready for use by all who desire enlightenment. We make use of the "irrigation treatment" in our daily practice, and are prone to confess it is the method *par excellence*.

The volume is gotten up in Messrs. Wood & Co.'s usual good style, and is one of the most practical and valuable volumes received for the reviewer, and we recommend it to the careful consideration of any physician who has a case of gonorrhœa for treatment.

---

FOURTEENTH YEAR.—Sixth revised edition Polk's Medical and Surgical Register of the United States and Canada. 2,500 pages, 6x9 inches, substantially bound, will be published early in 1900. This is positively the only national medical directory published. Embraces names of over 130,000 physicians, with college graduation, list of medical colleges, societies, boards of health, medical journals, mineral springs, hospitals, sanitariums, asylums and other medical institutions; also medical laws of each State.

From Aetna Life Insurance Company, medical department, Hartford, Conn.:

"We would be willing to pay more for it if we could have it oftener. * * * We notice from time to time an error, but suppose that can hardly be obviated in a work so large. We think physicians are going to be more particular to see that they answer inquiries regarding graduation, etc. We have refused to consider many during the past few years because their names were not in the register, and if so, their record was not complete."

Physicians who have not given their names to canvassers for insertion in the register are requested to send to R. L. Polk & Co., Detroit, Mich., immediately. Subscription price per copy, $10.00. R. L. Polk & Co., publishers, Detroit, Mich.

---

SAUNDERS' MEDICAL HAND ATLASES.—ATLAS OF DISEASES OF THE SKIN, including an epitome of Pathology and Treatment. By Prof. Dr. Franz Mracek, of Vienna. Authorized translation from the German. Edited by Henry W. Stelwagon, M. D., Ph. D., Clinical Professor of Dermatology, Jefferson Medical College, Philadelphia. Sixty-three colored plates and thirty-nine full-page half-tone illustrations. Pages 199. Price, $3.50, cloth, net. Philadelphia: W. B. Saunders, 925 Walnut Street. 1899.

To understand skin diseases one must have advantage of clinical observation, but this desideratum cannot come to all as skin clinics of much size are only to be had in the large cities. In the absence of clinical material this atlas is a most satisfactory substitute, as it presents by its numerous illustrations the most accurate reproductions of clinic work as interpreted by the best clinical teachers. The illustrations do not consist of the more rare forms of skin disease, but are for the most part representations of the more common diseases. It is a work that should appeal to the general practitioner. Judging from the demand for these atlases, the publisher has struck a bonanza. The contract for the American edition was 100,000

copies, but up to May last the expectation was nearly double that number.

T. J. B.

---

The Newer Remedies.—Including their synonyms, sources, methods of preparation, tests, solubilities, incompatibles, medical properties, and doses as far as known, together with sections on organic therapeutic agents and indifferent compounds of iron. A reference manual for physicians, pharmacists and students. By Virgil Coblentz, A. M., Phar. M., Ph. D., F. C. S., etc., Professor of Chemistry and Physics in the New York College of Pharmacy, etc. Third edition. Revised and very much enlarged. Pages, 147. Price, $1.00. Philadelphia: P. Blakiston's Son & Co., 1012 Walnut Street. 1899.

The author in the preface says: "If we consider the introduction of Kairin in 1882 as the beginning of the era of modern synthetic medicine, and that up to 1896 such products, in addition to various proprietary combinations of similar character, numbered about 800, with a further addition of about 1200 during the last three years, the future of this subject appears to be entirely beyond all conjecture."

All of the newer medicines are listed in this book, with a brief description, and as far as possible their chemical formula. The book is valuable as a work of reference especially for pharmacists and students.

T. J. B.

---

Refraction and How to Refract. Including Sections of Optics, Retinoscopy, the Fitting of Spectacles and Eye Glasses, etc. By James Thorington, A. M., M. D., Adjunct Professor of Ophthalmology in the Philadelphia Polyclinic and College for Graduates in Medicine; Assistant Surgeon at Wills' Eye Hospital; Associate Member of the American Ophthalmological Society; Fellow of the College of Physicians of Philadelphia; Member of the American Medical Association; Ophthalmologist to the Elwyn and the Vineland Training Schools for Feeble-minded Children; Resident Physician and Surgeon Panama Railroad Co., at Colon (Aspinwall), Isthmus of Panama, 1882-1889, etc. Two hundred illustrations, thirteen of which are colored. Octavo. 301 pages. $1.50 net, cloth. P. Blakiston's Son & Co., 1012 Walnut Street, Philadelphia, Pa. 1900.

Those whose good fortune it has been to attend a post-graduate course in the Philadelphia Polyclinic will remember with what painstaking care and ability Professor Thorington taught refractions, and will receive his book with open arms. Refraction has been made possible by men who have a limited knowledge of mathematics and who would literally fail over Donder's classic treatise. It is an excellent work on refraction and should be popular.

